# Supplementary material for: Diversity of Bacteria with Quorum Sensing and Quenching Activities from Hydrothermal Vents in the Okinawa Trough
Source: Microorganisms. 2023 Mar 14;11(3):748. doi: 10.3390/microorganisms11030748 (PMC10052519; doi:10.3390/microorganisms11030748)
Supplement: Supplementary file 1 [file microorganisms-11-00748-s001.zip › microorganisms-2194710-supplementary/Supplementary material/Supplementary Information.pdf]

Supplementary material for

Diversity of Bacteria with Quorum Sensing and Quenching Activities from Hydrothermal Vents in the Okinawa Trough

Fu Yin [1,2], Di Gao [1], Li Yue [1], Yunhui Zhang [1,3], Jiwen Liu [1,2,3], Xiao-Hua Zhang [1,2,3] and Min Yu [1,2,3,*]

[1] Frontiers Science Center for Deep Ocean Multispheres and Earth System, College of Marine Life Sciences, Ocean University of China, 5 Yushan Road, Qingdao 266003, China
[2] Laboratory for Marine Ecology and Environmental Science, Laoshan Laboratory, Qingdao 266237, China
[3] Institute of Evolution & Marine Biodiversity, Ocean University of China, Qingdao 266003, China
* Correspondence: yumin@ouc.edu.cn; Tel./Fax: +86-532-82032721

Abbreviations:

QS, quorum sensing.

One figure were shown in the supplemental material.

Citation: Yin, F.; Gao, D.; Yue, L.; Zhang, Y.; Liu, J.; Zhang, X.-H.; Yu, M. Diversity of Bacteria with Quorum Sensing and Quenching Activities from Hydrothermal Vents in the Okinawa Trough. *Microorganisms* **2023**, *11*, x. https://doi.org/10.3390/microorganisms11030748

Academic Editor: Giuseppina Tommonaro

Received: 16 January 2023
Revised: 9 March 2023
Accepted: 10 March 2023
Published: 14 March 2023

Copyright: © 2023 by the authors. Submitted for possible open access publication under the terms and conditions of the Creative Commons Attribution (CC BY) license (https://creativecommons.org/licenses/by/4.0/).

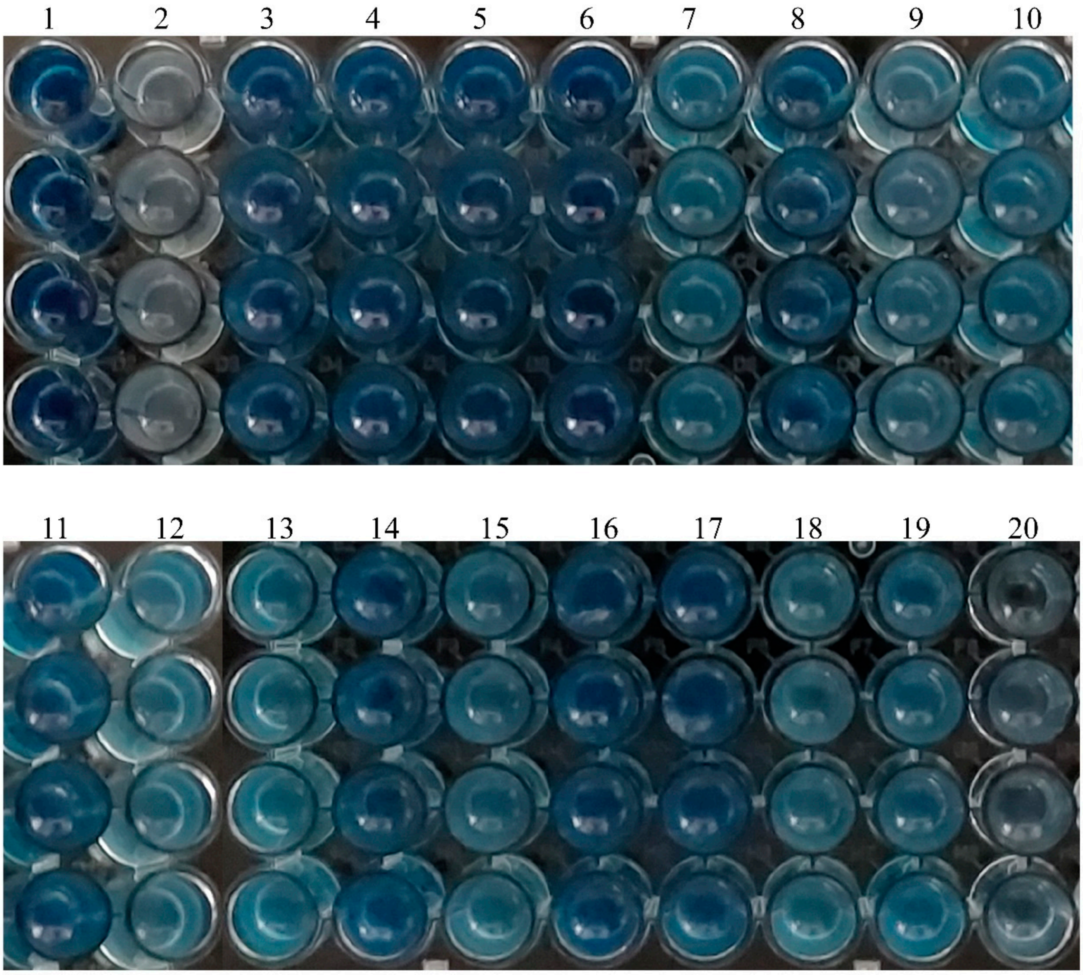

Figure S1. The AHL-producing abilities of strains are tested by the high-throughput method. The results are shown via the high-throughput method supplemented with the AHL reporter strain *Agrobacterium tumefaciens* A136. The wells 1–20 represent the following samples, in order: the positive control (C6-HSL 10 nM), the negative control (MB medium), *Stakelama pacifica* LLJ869, *Pseudohoeflea suaedae* SCR2, *Thalassococcus profundi* RWAS1, *Nitratireductor indicus* LLJ939, *Cellulomonas taurus* BOS2, *Klebsiella michiganensis* BODM11, *Brachybacterium muris* LLJ752, *Marinobacter zhanjiangensis* RWCR7, *Enterobacter hormaechei* BOM1, *Sphingobium yanoikuyae* RASR5, *Martelella mediterranea* LLJ1022, *Yoonia rosea* YESM7, *Cyclobacterium marinum* CCS19, *Roseovarius indicus* CCR3, *Microvirga calopogonii* CCM21, *Arenibacter palladensis* CCM2, *Neobacillus niacini* CJG092, *Paracoccus rhizosphaerae* CJG283.
